# Seasonal pattern of *Echinococcus* re-infection in owned dogs in Tibetan communities of Sichuan, China and its implications for control

**DOI:** 10.1186/s40249-016-0155-4

**Published:** 2016-07-05

**Authors:** Qian Wang, Wen-Jie Yu, Bo Zhong, Jing-Ye Shang, Liang Huang, Alexander Mastin, Renqingpengcuo, Yan Huang, Guang-Jia Zhang, Wei He, Patrick Giraudoux, Wei-Ping Wu, Philip S. Craig

**Affiliations:** Sichuan Provincial Center for Diseases Control and Prevention, Chengdu, Sichuan China; School of Environment and Life Sciences, University of Salford, Greater Manchester, M5 4WT UK; Shiqu County Center for Diseases Control and Prevention, Sichuan, China; Department of Chrono-environment, UMR UFC/CNRS 6249 aff. INRA, Université de Franche-Comté, Besançon, France; Institute of Parasitic Diseases, China Center for Diseases Control and Prevention, Shanghai, China

**Keywords:** *Echinococcus*, Dog infection, Seasonal pattern, Tibetan communities, China

## Abstract

**Background:**

Human cystic echinococcosis (CE) and alveolar echinococcosis (AE) are highly endemic in Tibetan communities of Sichuan Province. Previous research in the region indicated that domestic dog was the major source of human infection, and observations indicated that domestic dog could have more access to intermediate hosts of *Echinococcus* spp.: both domestic livestock (CE) viscera and small mammals (AE), in early winter and again in spring. We hypothesized that there would therefore be a significant increase in the risk of canine infection with *Echinococcus* spp. in these two seasons and conducted a reinfection study to investigate this further.

**Methods:**

Faecal samples were collected from owned dogs in seven townships in Ganze Tibetan Autonomous Prefecture (Sichuan Province, China), and *Echinococcus* spp. infection status was determined using copro-antigen ELISA. Dogs were sampled in April (spring), July (early summer), September/October (autumn/early winter) and December (winter) in 2009; and in April (spring) 2010. Dogs were treated with praziquantel following each of the five sample collections to eliminate any tapeworms. Information on dog sex, age and body weight was also collected. The t-test, Fisher’s exact test, Poisson regression and logistic regression were used to compare means and prevalences, and to identify factors associated with infection status.

**Results:**

The proportion of female dogs was significantly lower than that of male dogs; female dogs had significantly higher (22.78 %) baseline copro-ELISA prevalence than males (11.88 %). Dog body weight, sex, age, county and previous infection status at any sampling point had no influence on the re-infection prevalence in general. Poisson regression did not found a significant influence on the re-infection prevalence due to different deworming/sampling time spans. Dogs exhibited significantly higher re-infection prevalences in spring and early summer of 2009 and in early winter between September/October and December of 2009, suggesting a higher infection pressure in these seasons comparing with other seasons.

**Conclusion:**

Following praziquantel treatment, dog body weight, sex, age, county, deworming time span and previous infection status at any sampling point had no influence on the re-infection prevalence in the region in general. The differences between re-infection prevalences were probably due to the seasonality in *Echinoccocus* spp. infection pressure in the region. Early winter, spring and early summer should be important seasons for optimal dog deworming intervention in these Tibetan communities.

**Electronic supplementary material:**

The online version of this article (doi:10.1186/s40249-016-0155-4) contains supplementary material, which is available to authorized users.

## Multilingual abstracts

Please see Additional file 1 for translations of the abstract into the six official working languages of the United Nations.

## Background

Human Cystic echinococcosis (CE) and the more pathogenic alveolar echinococcosis (AE) are caused by infection with the larval stages of tapeworms of *Echinococcus granulosus* and *E. multilocularis*, respectively, after accidental ingestion of eggs. The eggs of both species may be shed in the faeces of dogs (or other canids) harbouring adult stages of these small tapeworms. *E. granulosus* has a global distribution while *E. multilocularis* occurs only in the northern hemisphere [1]. In humans, lesions develop primarily in the liver. AE is one of the most lethal zoonotic parasitic diseases, with a high mortality rate if untreated [2, 3]. The World Health Organization (WHO) listed echinococcosis as a Neglected Tropical Disease in 2010 [4]. Human echinococcosis (both CE and AE) is highly endemic in western China, where pastoralism is predominant and overall the socio-economic development level is much lower than that of eastern China. The Chinese Ministry of Health has estimated that around 380 000 echinococcosis patients were present in the western region in 2004 [5]. The estimated worldwide human burden of CE is 285,407 DALYs (95 % confidence interval [*CI*], 218 515–366 133) [6], and the figure for AE is 666 434 DALYs (95 % *CI* 331 000–1.3 million) [7]. China is reported to be responsible for 40 % of the global CE burden [6] and 91 % of global AE burden [7]. Thus, echinococcosis is one of the most important infectious diseases, zoonosis and public health problems in rural communities of western China.

Western Sichuan Province is located in the south-eastern part of the Qinghai-Tibet Plateau, with an elevation above 3 500 m and a population of some 2 million Tibetans. This area has been previously identified as highly endemic for both human AE and CE [8, 9]. Dogs were indicated to be the major zoonotic transmission source for both diseases in western China [10–13], although foxes, including the Tibetan fox (*Vulpes ferrilata*), are considered to be a major wildlife reservoir for *E. multilocularis* in northwest Sichuan [14].

In the Tibetan communities of western Sichuan Province, human settlements can be broadly classified as towns, farms or high pastures (>3 500 m). Keeping livestock such as yaks and/or sheep and goats is very common in the area, and domestic dog has an important role in the guarding of households, tents and livestock. The infrastructure in the Tibetan region is underdeveloped, which makes public service delivery, including medical and veterinary services, very challenging. There are few slaughterhouses and people usually slaughter their livestock near their tents/houses in early winter [15]. The natural mortality of the livestock (and wild ungulate) intermediate hosts of *E. granulosus* may also be high in early spring due to extreme weather conditions and shortage of forage [16]. Based upon this, it has been hypothesized that dogs would have a higher probability of access to viscera of livestock (and wild ungulates) which might have CE cysts in early winter and spring [13, 17, 18]. Regarding *E. multilocularis*, high population densities of the small mammal intermediate hosts of this parasite*,* such as the plateau pika (*Ochotona curzoniae*) and Qinghai vole (*Microtus fuscus*), have been observed in early spring [19, 20]. These high densities may increase the potential for predation by dogs and subsequent exposure to *E. multilocularis* during this season.

Despite the potential for seasonality in *Echinoccocus* spp. infection pressure for dogs, little work has been undertaken to investigate this further. The current report describes an attempt to quantify this seasonality by measuring the rate of re-infection of dogs with *Echinococcus* spp. in Tibetan communities following praziquantel dosing, using a commercially available copro-ELISA test.

## Methods

### Site description

The study was carried out over a 12 month period (from April 2009 to April 2010) in Shiqu and Seda counties of Ganzi Tibetan Autonomous Prefecture in northwest Sichuan Province, China. The selection of the target communities was based on a documented high prevalence of human echinococcosis from mass ultrasound screening [8]. The overall human echinococcosis prevalence within Shiqu and Seda counties was reported to be 12.09 % (with CE prevalence of 7.46 % and AE prevalence of 4.67 %) and 6.30 % (with CE prevalence of 4.48 % and AE prevalence of 2.02 %), respectively [9].

Shiqu County (area 25 191 km^2^, mean elevation 4 200 m) is located on the eastern Qinghai-Tibet Plateau (97°20′-- 99°15′E and 32°19′-- 34°20′N), and shares a border with Qinghai Province in the east, north and west and with the Tibet Autonomous Region in the south. It had a human population of 86 800 in 2012 and around 75 % of the area was used as grazing pasture [21]. Seda County (area 9 339 km^2^, mean elevation 4 127 m) is also located on the eastern Qinghai-Tibet Plateau (98°48′- 101°00′E and 31°38′- 33°20′N) and shares a border with Qinghai Province in the north and with Aba Qiang Nationality and Tibetan Autonomous Prefecture in the east. It had a population of 45 660 in 2012 and approximately 80 % of the area can be used as grazing pasture [21]. The general climatic conditions in both areas are similar [15].

### Collection and examination of faecal samples

**Fig. 1:**
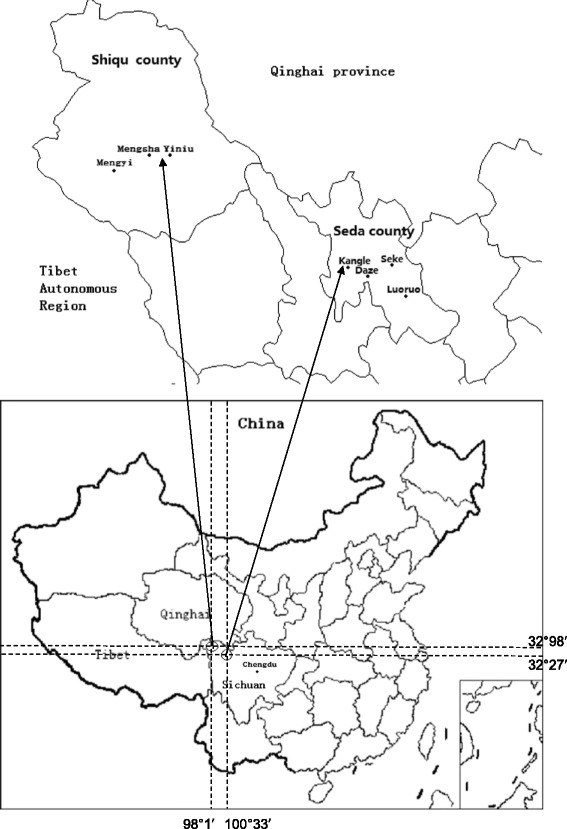
Study areas of Shiqu and Seda counties in Sichuan Province, China

The study was approved by the Ethical Committee of Sichuan Provincial Center for Diseases Control and Prevention. The study performed five rounds of canine praziquantel treatment and faecal sample collection in three townships of Shiqu County (Mengyi, Yiniu and Mengsha) and 4 townships of Seda County (Kangle, Seke, Daze and Luoruo) (See Fig. 1). Townships were visited in April, July, September, October and December 2009, and in April 2010. Before going to the field, researchers were trained on how to collect both canine faecal samples and other relevant information regarding the dogs sampled: sex was confirmed directly from owners; age was assessed by tooth growth; and body weight was approximated by visual assessment. In each sampling, all accessible owned dogs over three months of age were registered with the owner’s permission. Faecal samples were collected from registered dogs before dosing the dogs with praziquantel tablets under supervision of the researchers. Praziquantel tablets were wrapped up in a ball of *tsampa* (traditional Tibetan ‘porridge’ made from barley) in order to increase compliance and were administered at the recommended dose of 5 mg/kg (reported 99.9 % efficacy) [22]. Faecal samples were collected from the ground used by tethered dogs. All faecal samples were tested using a commercial copro-ELISA kit (Zhuhai Haitai Bio-Pharmaceutical Co. Ltd., Zhuhai, Guangdong, China) in order to estimate the *Echinococcus* spp. coproantigen prevalence. The sensitivity and specificity of the test for detection of *Echinococcus* spp. was reported by a national test organized by the China for Disease Control [23] to be 83.9 and 74.7 % respectively.

### Data analysis

All data were initially entered into a Microsoft Excel document (Microsoft, Redmond, WA, USA), and were subsequently analysed using the statistical analysis package R (R Foundation for Statistical Computing, Vienna, Austria). The age and body weight of the registered dogs were described using the means and standard deviation (SD), and the proportion of female dogs was estimated. 95 % confidence interval (95 % *CI*) were presented for the means and proportions, and differences in mean age and mean body weight of dogs between groups were investigated using the t-test. Fisher’s exact test was used to compare the copro-ELISA prevalence between sampling time points.

Poisson regression used to investigate whether the different time-span between sampling points was associated with the re-infection prevalence. The Poisson regression model adopted a log link; the dependent variable of the Poisson regression model was the number of positive samples; the offset was the log of the number of samples tested; and the independent variable was time-span. Finally, stepwise logistic regression analysis was used to identify factors (i.e. county, sex, body weight, dosage, and previous infection status) influencing the copro-prevalence of each visit. Separate analyses were conducted for two formulations of the data: one including all available dogs registered initially (study A), and one including only those dogs sampled at all sampling points (study B). Results of statistical tests were classed as significant at a *P* < 0.05 level.

## Results

The sample size at each visit for study A was 584, 328, 141, 99 and 64 during April, July, September/October, and December, 2009; and April, 2010, respectively. The sample size for study B was 64 at all sampling points.

### Dog populations

At the time of enrolment into the study, the mean age amongst dogs in study A was 4.33 years (95 %*CI* = 4.13–4.53, SD = 2.39), and the mean body weight was 15.64 kg (95 %*CI* = 15.13–16.16, SD = 6.22). For Study B, the mean age at enrolment was 4.67 years (95 %*CI* = 3.40– 5.35, SD = 2.56), and the mean body weight was 14.03 kg (95 %*CI* = 12.88– 15.19, SD = 4.39). The proportion of females in study A was 13.40 %, and that in study B was 14.06 %. There was no evidence of a difference in mean age and body weight between the two groups (t-test *P* > 0.05), or in the proportion of females (Fisher’s exact test *P* > 0.05).

### Copro-prevalence levels

For study A, the *Echinococcus* spp. copro-prevalence (hereafter referred to as ‘prevalence’) at the first visit in April 2009 (the baseline) was 13.36 % (78/584) (Table 1). The prevalence at the sampling point in July 2009 was significantly lower than this prevalence (5.8 %, *P* < 0.001), and a significant decrease from this prevalence was observed at the September/October 2009 visit (0.0 %, *P* = 0.001). At the visit in December 2009, the prevalence was found to be significantly higher than that two months previously (4.04 %, *P* = 0.028). No significant difference was found between the two prevalences in December 2009 and April 2010.

**Table 1:** Comparing the prevalences between dog groups and between neighbouring sampling points by Fisher’s exact test

Month/year	Study A		Study B		*P* value for comparing prevalences between the two groups
	Prevalence (No.positive/No.tested)	*P* value for comparing a prevalence with its immediate previous prevalence	Prevalence (No.positive/No.tested)	*P* value for comparing a prevalence with its immediate previous prevalence	
Apr.,2009	13.36 % (78/584)		12.5 % (8/64)		1
Jul.	5.8 % (19/328)	<0.001	3.13 % (2/64)	0.096	0.549
Sept./Oct.	0.0 % (0/141)	0.001	0.0 % (0/64)	0.496	1
Dec.	4.04 % (4/99)	0.028	4.69 % (3/64)	0.244	1
Apr.,2010	1.56 % (1/64)	0.649	1.56 % (1/64)	0.619	1

**Fig. 2:**
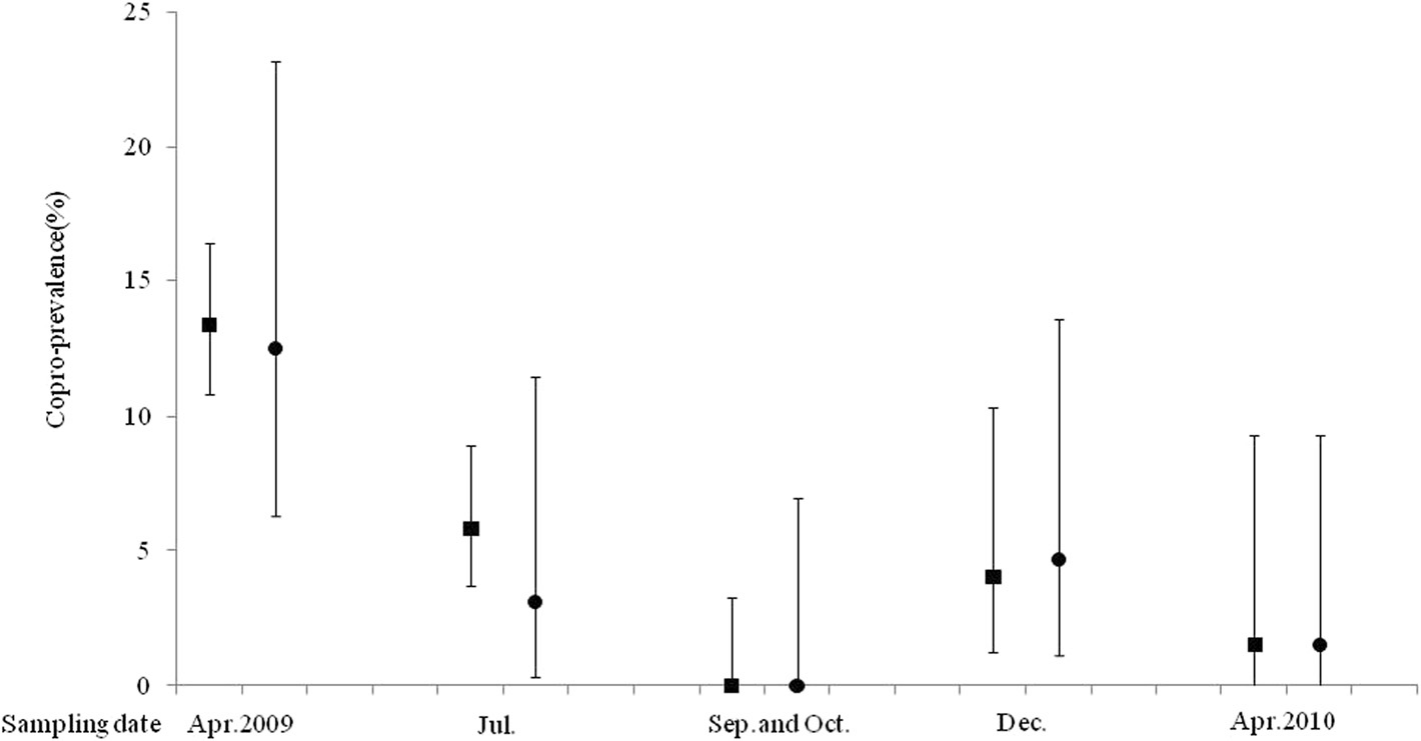
The 95 %CI of dogs’ copro-prevalences for Study A and B at the five sampling points. ■ Study A ● Study B

The general pattern of change in prevalence for study B was similar to that of study A, but consecutive differences were not found to be statistically significant (see Table 1). There was no significant difference in prevalence between Study A and Study B for any of the five samplings (See Table 1 and Fig. 2).

### Factors influencing copro-prevalence

For Study A, multivariable analysis of risk factors for ELISA positivity at each visit point identified county and sex to be associated with ELISA status during the baseline visit (April 2009) see Table 2. At this point, the prevalence in Seda was 18.25 %, whereas that in Shiqu was 9.34 %. The prevalence amongst females at this same visit was 22.78 %, whereas that in males was 11.88 %. County was found to be a significant factor at the sampling point in July 2009, where the prevalence in Seda was 13.22 % compared to 1.45 % in Shiqu (Table 3). No significant factors were identified for the subsequent three re-infection prevalences (see Table 2).

**Table 2:** Factors influencing the prevalence of *Echinococcus* spp. infection in dogs in Study A

Month/year	Factors	Coefficient	S.E.	Wald	df	*P*	Odds ratio (95 %*CI*)
2009							
Apr.(baseline)	Shiqu vs. Seda	−0.86	0.25	11.48	1	0.001	0.42 (0.26–0.70)
	Female vs. male	0.86	0.31	7.75	1	0.005	2.36 (4.33–1.29)
	Constant	−1.57	0.17	81.73	1	<0.001	0.21 (0.15–0.29)
Jul.	Shiqu vs. Seda	−2.34	0.64	13.32	1	<0.001	0.10 (0.03–0.34)
	Constant	−1.87	0.27	48.58	1	<0.001	0.15 (0.09–0.26)

**Table 3:** Copro-Elisa prevalences for Study A and B in Shiqu and Seda counties respectively

Month/year	Study A			Study B		
	Prevalence for Shiqu county (No.positive/No.tested)	Prevalence for Seda county (No.positive/No.tested)	*P* value for comparing the prevalences between two counties	Prevalence for Shiqu county (No.positive/No.tested)	Prevalence for Seda county (No.positive/No.tested)	*P* value for comparing the prevalences between two counties
Apr.,2009	9.34 % (30/321)	18.25 % (48/263)	0.002	13.21 % (7/53)	9.09 % (1/11)	1
Jul.	1.45 % (3/207)	13.22 % (16/121)	<0.001	1.89 % (1/53)	9.09 % (1/11)	0.316
Sept./Oct.	0 % (0/108)	0 % (0/33)	1	0 % (0/53)	0 % (0/11)	1
Dec.	4.11 % (3/73)	3.85 % (1/26)	1	3.77 % (2/53)	9.09 % (1/11)	0.438
Apr.,2010	1.90 % (1/53)	0 % (0/11)	1	1.89 % (1/53)	0 % (0/11)	1

For study B, no factors were found to be associated with the prevalence at any sampling point (*P* > 0.05). Fisher’s exact test also did not find any difference in prevalence between the two counties at any sampling point for this study (Table 3).

There was no evidence that the reinfection prevalence was associated with the previous infection status for both studies, as assessed using stepwise logistic regression (*P* > 0.05). Poisson regression did not found a significant influence (*P* > 0.05) on the re-infection prevalence due to different deworming/sampling time spans for both studies.

## Discussion

Owned dogs are considered to be most important zoonotic transmission source for both human cystic echinococcosis (CE) and alveolar echinococcosis (AE) on the Qinghai-Tibet Plateau [10, 11]. A previous study in Shiqu County showed that the faeces of owned dogs were mainly distributed around the houses of dog owners (proximity 0–200 m) [24]. Faeces from owned dogs that were PCR positive for *E. multilocularis* DNA were also shown to be spatially clustered [25]. Control of echinococcosis in dogs is therefore an integral component of public health programme aiming to control human echinococcosis in the region.

A strategy for sustainable control of canine echinococcosis should ideally be based on an understanding of dog re-infection patterns, especially in areas co-endemic for CE and AE [26]. A single oral administration of praziquantel (5.0 mg/kg bw) to dogs was found to be 99.9 % effective against both *E. granulosus* and *E. multilocularis* [22, 27, 28]. Current *Echinococcus* spp. copro-antigen ELISA tests are genus specific up to 95 % and thus provide a useful tool to measure prevalence [29]. Moss et al. [30] used copro-ELISA and copro-PCR to determine re-infection of owned dogs in Shiqu County after a single dose of praziquantel in May 2006. The study found an average copro-ELISA prevalence of 10 % two months after this treatment (July 2006); 3 % five months after (October 2009); and 11 % one year after (May 2007). Despite not using repeated dosing as in the current study, these results are suggestive of a lower infection pressure in summer and autumn and higher infection pressure in winter and early spring. The current research was designed to assess overall *Echinococcus* spp. re-infection patterns of dogs in Tibetan communities in two counties (Shiqu and Seda) in Sichuan Province over a 12 month period (April 2009 to April 2010) with particular attention paid to potential seasonal variation. As such, all dogs were treated with praziquantel at each gsampling round in order to ensure that any infections detected at the subsequent round from dosed dogs occurred during the period between treatment and sampling. The current study also found some evidence of a lower re-infection pressure in summer and autumn, and a higher re-infection pressure in spring. In addition, it revealed a more specific higher re-infection pressure season of early winter (from October to December). The current study did not find an influence on the seasonality of re-infection by different deworming/sampling time spans for both studies. Early winter is the main slaughtering season in the region, where the prevalence of livestock CE has been estimated to be between 7.3 and 76 % for yaks [31–35]; 14 and 82 % for sheep; and 3.5 and 48 % for goats [36]. The current study therefore suggests that intensive praziquantel treatment of dogs (preferably monthly dosing) would be advantageous in early winter, spring and early summer.

Possible death of intermediate hosts of *E. granulosus* due to extreme cold and forage shortage in winter and early spring was assumed to be an important risk factor for the prevalence of CE on the plateau [9, 17, 31]. These hosts include wild ungulates and livestock (yaks, sheep and goats). Wild ungulates have been found to have a prevalence of CE, with estimates of 6.42 % (21/327) amongst blue sheep (*Pseudois nayaur*) and 6.57 % (13/198) for Tibetan gazelle (*Procapra picticaudata*) in the Qinghai part of the Plateau [37]. Therefore, it was considered that a higher opportunity for exposure/re-infection of *E. granulosus* in dogs could occur in winter and early spring. However, the current study did not support the assumption of a higher exposure in the period between December 2009 and April 2010, which is the winter and early spring in the region. No report of extreme cold and forage shortage in the period might explain why the assumption was not supported.

Several small mammal species, such as the Qinghai vole (*Microtus fuscus*) and the Plateau pika (*Ochotona curzoniae*), are known to act as intermediate hosts for *E. multilocularis*, with the plateau pika considered to be of particular importance [38, 39]. The plateau pika is known to be diurnal, with most activity observed between 06:30 and 20:00 between January and April (based upon field investigations in January, April, August, October and November) [19, 20]. Owned tethered dogs were usually released at around 20:00 and leashed again at around 08:00 the following morning – providing these dogs with opportunities to prey on small mammals with nocturnal and/or crepuscular activity [24, 30]. The current study found that the highest re-infection prevalence occurred in July 2009 (for study A), following dosing in April, which suggests that the highest exposure to *Echinococcus* spp. occurred between these two months. This may be associated with greater opportunities for access to small mammal hosts during spring and early summer.

A preference for keeping male dogs in Tibetan communities has been observed in other surveys [30, 40], and the same pattern was found in the current study. Despite female dogs being less commonly kept than male dogs (amongst owned dogs), a significantly higher *Echinococcus* spp. copro-prevalence was observed amongst female dogs than male dogs at the first sampling (April 2009). The reason behind this higher prevalence in female dogs is unknown. It is also not clear why the prevalence in Seda county were higher than those in Shiqu county during the first two rounds of sampling points (April and July 2009) (Table 3). These differences were not apparent in subsequent visits.

The *Echinococcus* re-infection prevalence was not found to be associated with the previous infection status at any sampling point. The prevalence (1.56 % for both study populations) at the sampling point in April 2010 were significantly lower than those in April 2009 (13.36 % for study A and 12.5 % for study B) (Fig. 2). The two results might imply the re-infection was largely determined by the existing reservoir of the parasite in hosts and the availability of the infectious viscera of intermediate hosts. Thus, it could be assumed that the owned dogs’ role as definite host to influence the reservoir was reduced drastically during the deworming. Therefore, it demonstrates the effect of repeated praziquantel dosing on reducing the prevalence of infection amongst owned dogs.

Stray dogs were not included in the current study. Although the stray dog population density was not found to be associated with the prevalence of human echinococcosis in a review of literature published between January 2000 and July 2011 [13], the role of stray dogs in the life cycle is not fully understood in the area. Further work investigating levels of infection amongst stray dogs would therefore be useful. Also, the number of dogs sampled decreased in each of the five sampling periods for Study A. This is partly associated with the Tibetan nomadic life style, where people move with their livestock (yaks and sheep) and dogs to higher pastures between May and October. Future research to measure the re-infection of owned dogs in summer pastures would be useful.

Regular deworming of all owned dogs is very hard to apply and sustain in the vast, remote, high altitude and difficult terrain of eastern Qinghai-Tibet Plateau. A New Zealand backed pilot intervention project in Garze County for cystic echinococcosis which included livestock vaccination, was not very successful in large part due to the logistics of dog deworming in the isolated area [41]. There is therefore a need for a simpler and more sustainable deworming strategy. It has been argued that effective dosing of owned dogs 2–4 times per year could have a major impact on both zoonotic risk and transmission potential for both *E. multilocularis* and *E. granulosus* in Tibetan communities [30]. One of the criteria defined by WHO for effective control of human CE as a public health problem, is to reduce the canine echinococcosis prevalence to 0.01 % [42]. The current study demonstrates that the canine prevalence could not be reduced to this level within one year by with four doses of praziquantel. This is likely due to the reservoir of infection in intermediate hosts, and suggests that higher dosing frequencies may be required to achieve low prevalence. The current study also suggests that ‘targeted’ anthelmintic dosing of dogs during the spring and early winter could be beneficial.

## Conclusion

Following praziquantel treatment, dog body weight, sex, age, county, deworming time span and previous infection status at any sampling point had no influence on the re-infection prevalence in the region in general. The differences between re-infection prevalences were probably due to the seasonality in *Echinoccocus* spp. infection pressure in the region. Early winter, spring and early summer should be important seasons for optimal dog deworming intervention in these Tibetan communities.

## Additional file

Additional file 1: Multilingual abstracts in the six official working languages of the United Nations. (PDF 595 kb)

## References

[1] Vuitton DA, Zhou H, Bresson-Hadni S, Wang Q, Piarroux M, Raoul F, Giraudoux P (2003). Epidemiology of alveolar echinococcosis with particular reference to China and Europe. Parasitology.

[2] Ammann RW, Eckert J, Thompson RCA, Lymbery AJ (1995). Clinical diagnosis and treatment of echinococcosis in humans. In *Echinococcus* and hydatid disease. *Echinococcus* and hydatid disease.

[3] Ammann RW, Eckert J (1996). Cestodes. Echinococcus. Gastroenterol Clin North Am.

[4] World Health Organization. Working to overcome the global impact of neglected tropical diseases, First WHO report on neglected tropical diseases. Geneva: WHO; 2010. p. 107–12.

[5] Ministry of Health (2008). Report on the national survey of current status of major human parasitic diseases in China.

[6] Budke CM, Deplazes P, Torgerson PR (2006). Global socioeconomic impact of cystic echinococcosis. Emerg Infect Dis.

[7] Torgerson PR, Keller K, Magnotta M, Ragland N (2010). The Global Burden of Alveolar Echinococcosis. PLoS Negl Trop Dis.

[8] Li TY, Qiu JM, Yang W, Craig PS, Chen XW, Xiao N, Ito A, Giraudoux P, Mamuti W, Yu W, Schantz PM (2005). Echinococcosis in Tibetan populations, Western Sichuan Province, China. Emerg Infect Dis.

[9] Wang Q, Huang Y, Yi DY, Huang L, Yu WJ, He W, Shang JY. Report on investigation of epidemic status of echinococcosis in Sichuan Province, China. Chengdu of Sichuan: Sichuan Provincial Center for Diseases Control and Prevention; 2014. p. 47.

[10] Craig PS, Giraudoux P, Shi D, Bartholomot B, Barnish G, Delattre P, Quere JP, Harraga S, Bao G, Wang Y, Lu F, Ito A, Vuitton DA (2000). An epidemiological and ecological study of human alveolar echinococcosis transmission in south *Gansu*. Chin Acta Trop.

[11] Wang Q, Qiu JM, Schantz P, He JG, Ito A, Liu FJ (2001). Investigation of risk factors for development of human hydatidosis among households raising livestock in Tibetan areas of western Sichuan province. Chin J Parasitol Parasit Dis.

[12] Wang Q, Qiu JM, Yang W, Schantz PM, Raoul F, Craig PS, Giraudoux P, Vuitton DA (2006). Socioeconomic and behavior risk factors of human alveolar echinococcosis in Tibetan communities in Sichuan, People’s Republic of China. Am J Trop Med Hyg.

[13] Wang Q, Huang Y, Huang L, Yu W, He W, Zhong B, Li W, Zeng X, Vuitton DA, Giraudoux P, Craig PS, Wu WP (2014). Review of risk factors for human echinococcosis prevalence on the Qinghai Tibet Plateau, China: a prospective for control options. Infect Dis Poverty.

[14] Jiang WB, Liu N, Zhang GT, Renqing PC, Xie F, Li TY, Wang ZH, Wang XM (2012). Specific detection of *Echinococcus* spp. from the Tibetan fox (*Vulpes ferrilata*) and the red fox (*V. vulpes*) using copro-DNA PCR analysis. Parasitol Res.

[15] Editorial Commission of Shiqu County Record (2000). Shiqu County Record 1997.

[16] Xinhua news. Early snow storm and low temperature, gazelles experience hardship this winter. http://www.sc.xinhuanet.com/content/2008-11/20/content_14971000.htm. Accessed 5 May 2014. 2008.

[17] Wang H, Ma SM, Cao DP, Zhao HL (2000). An epidemiology survey on human hydatidosis in Southern Qinghai Plateau. Chin J Parasit Dis Control.

[18] He DL (2000). Endemic of echinococcosis and its control in Qinghai Province. Chin J Zoonoses.

[19] Fan NC, Dou FM (1990). Observation of ground activities of Plateau Pika. J Zool.

[20] Qu KJ, Li KX, Yang M, Li WJ, Zhang YM, Smith AT (2007). Seasonal dynamic pattern of spatial territory in social groups of plateau pika (*Ochotona curzoniae*). Acta Theriol Sin.

[21] Sichuan Year Book. http://nj.sc.gov.cn/Yearbook2012.shtml. Accessed 8 May 2014. 2013

[22] WHO/OIE. Manual on Echinoccosis in Humans and Animals: a Public Health Problem of Global Concern. Eds Eckert J, Gemmell MA, Meslin F-X & Pawlowski ZS. Paris, France 2001: 265

[23] Wang LY, Wu WP (2010). Evaluation of coproantigen kits produced in China for dog echinococcus detection.

[24] Vaniscotte A, Raoul F, Poulle ML, Romig T, Dinkel A, Takahashi K, Giraudoux P (2011). Role of dog behaviour and environmental faecal contamination in transmission of *Echinococcus multilocularis* in Tibetan communities. Parasitology.

[25] Wang Q, Budke C, Huang L, Giraudoux P, Raoul F, Vuitton DA, Qiu DC (2012). Spatial clustering of *Echinococcus multilocularis* infected dogs in pastoral Tibetan Communities, Sichuan, China. J Prev Med Inform.

[26] Lembo T, Craig PS, Miles MA, Hampson KR, Meslin FX, Macpherson CNL, Meslin FX, Wandeler AI (2013). Zoonoses prevention, control, and elimination in dogs. Dogs, Zoonoses and Public Health.

[27] Oakley GA (1991). Anthelmintics for cats and dogs.

[28] Bauer C (1994). Anthelminthika zum Einsatz gegen Helminthen des Verdauungstraktes, der Atemwege und Harnblase von Hund und Katze-eine Uebersicht. Kleintierpraxis. Harnblase bei Hund und Katze – eine Übersicht. Kleintierpraxis.

[29] Allan JC, Craig PS (2006). Coproantigens in taeniasis and echinococcosis. Parasitol Int.

[30] Moss JE, Chen X, Li T, Qiu J, Wang Q, Giraudoux P, Ito A, Torgerson PR, Craig PS (2013). Reinfection studies of canine echinococcosis and role of dogs in transmission of *Echinococcus multilocularis* in Tibetan communities, Sichuan, China. Parasitology.

[31] He JG, Qiu JM, Liu FJ, Chen XW, Liu D, Chen WD (2000). Epidemiological survey on hydatidosis in Tibetan region of western Sichuan. II: Infection situation among domestic and wild animals. Chin J Zoonoses.

[32] Li WK, Zhang XL, Guo FC, Wu BT (2002). A review on livestock echinococcosis prevention and control strategy in Gansu province. Chin J Vet Parasitol.

[33] Liu GL, Hao YH (2002). Epidemiology of echinococcosis in Huangnan district, Qinghai province, China. Chin J VetParasitol.

[34] Ma XQ (2002). Echinococcosis in Yak population in Guomaying township, Guinan, Qinghai province, China. Chin Qinghai J Anim Vet Sci.

[35] Tan SK, Chen GL. Survey on yak and sheep hydatid disease in Gangcha district, Qinghai province, China. Chin Qinghai J Anim Vet Sci. 2005;35(2):21–1 (in Chinese).

[36] Wang ZH, Wang XM, Liu XQ (2008). Echinococcosis in China, a Review of the Epidemiology of *Echinococcus* spp. Ecohealth.

[37] Zhang JX, Wang H (2007). Epidemiological Survey on *Echinococcus* infection in animals in Qinghai Province. Chin J Parasitol Parasit Dis.

[38] Xiao N, Nakao M, Qiu J, Budke CM, Giraudoux P, Craig PS, Ito A (2006). Dual infection of animal hosts with different *Echinococcus* species in the eastern Qinghai-Tibet plateau region of China. Am J Trop Med Hyg.

[39] Ma J, Wang H, Lin G, Craig PS, Ito A, Cai Z, Zhang TZ, Han XM, Ma X, Zhang JX, Liu YF, Zhao YM, Wang YS (2012). Molecular identification of *Echinococcus* species from eastern and southern Qinghai, China, based on the mitochondrial cox1 gene. Parasitol Res.

[40] Bogel K. Guidelines for dog rabies control. http://www.who.int/rabies/animal/en/vph8343rev1.pdf. Accessed 25 June 2013.

[41] Craig PS, Larrieu E (2006). Control of cystic echinococcosis/hydatidosis: 1863–2002. Adv Parasitol.

[42] WHO (2011). Report of the WHO informal working group on cystic and alveolar echinococcosis surveillance, prevention and control, with the participation of the Food and Agriculture Organization of the United Nations and the World Organization for Animal Health.

