# Supplementary material for: Seasonal pattern of Echinococcus re-infection in owned dogs in Tibetan communities of Sichuan, China and its implications for control
Source: Infect Dis Poverty. 2016 Jul 5;5:60. doi: 10.1186/s40249-016-0155-4 (PMC4932717; doi:10.1186/s40249-016-0155-4)
Supplement: Additional file 1: — Multilingual abstracts in the six official working languages of the United Nations. (PDF 595 kb) [file 40249_2016_155_MOESM1_ESM.pdf]

Translation of the abstract into the five official working languages of the United Nations

لنمط الموسمي لعودة عدوى ديدان المشوّكات في الكلاب التي تقتنيها المجتمعات التبتية في مقاطعة سيشوان، الصين، ومقتضيات السيطرة عليها

كيان وانغ، وين-جي يو، بو زونغ، جنغ-يي شانغ، ليانغ هوانغ، الكساندر ماستن، RENQINGPENGCUO، يان هوانغ، جوانغ-جيا زانغ، وي هي، باتريك جيرودو، وي-بنغ وُو، فيليب س. كريغ.

خلاصة

خلفية: إنّ داء المشوّكات الكيسية (CE) وداء المشوّكات السنخية (AE) الذين يصيبان الإنسان متوطنان بشكل كبير في المجتمعات التبتية في مقاطعة سيشوان. أشار بحث سابق في المنطقة الى أنّ الكلاب الأهلية هي السبب الرئيس في اصابة البشر، كما وقد اشارت المراقَبات الى أنّ الكلاب الأهلية قد تملك فرصة وصول أكبر الى المضيف الوسيط لأنواع المشوّكات: أحشاء المواشي (CE) واللبائن الصغيرة (AE)، في بدايات الشتاء ومرة أخرى في الربيع. ولذلك نفترض أنّ مخاطر اصابة الكلاب بأنواع المشوّكات تزداد بصورة كبيرة في هذين الفصلين وأجرينا دراسة اعادة عدوى للتحقق من الأمر بشكل أكبر.

الطرائق: تمّ جمع عينات البراز من الكلاب المقتناة في سبع مدن في اقليم غانزي ذو الحكم الذاتي في التبت (سيشوان، الصين)، وتم تحديد حالة عدوى انواع المشوّكات باستخدام المستضدات البرازية بطريقة المُقايَسَةُ الامْتِصاصِيَّةُ المَناعِيَّةُ للإنْزيمِ المُرْتَبِط (إليزا). تم أخذ العينات من الكلاب في نيسان (الربيع)، تمّوز (مطلع الصيف)، أيلول، وتشرين الأول (الخريف، ومطلع الشتاء)، و كانون الأول (الشتاء) من عام 2009؛ وأيضا في نيسان (الربيع) 2010. تم علاج الكلاب بـ برازيكوانتيل عقِب كل واحدة من عمليات أخذ العينات للتخلص من الديدان الشريطية. كما وتمّ جمع معلومات عن أجناس الكلاب، وأعمارها، وأوزانها. تم استخدام اختبارات: اختبار تي، واختبار فيشر الدقيق، وانحدار بواسون والانحدار اللوجستي لمقارنة المعدلات ومعدلات الانتشار ولتحديد العوامل المصاحبة لحالة العدوى.

النتائج: كانت نسبة الكلاب الإناث أقلّ بصورة كبيرة من الذكور؛ كان للإناث قيمة قاعدية لفحص (إليزا) لانتشار العدوى عالية بشكل كبير (22.78%) مقارنة بالذكور (11.88%). لم يكن لأوزان الكلاب، اجناسها، اعمارها، المدينة التي جاءت منها، او الحالة المعدية السابقة في أي من مراحل أخذ العينات أي تأثير على معدل انتشار عودة العدوى عموماً. لم يكشف اختبار انحدار بواسون عن أي تأثير ملحوظ على معدل انتشار عودة العدوى بتأثير اختلاف الفترات الزمنية لطرد الديدان\أخذ العينات. أبدت الكلاب معدل انتشار عودة عدوى اكبر بكثير في ربيع ومطلع صيف 2009 وفي مطلع شتاء بين أيلول\تشرين الأول و كانون الأول 2009، مما يقترح زخماً أكبر للعدوى خلال هذه الفصول مقارنة بالفصول الأخرى.

الاستنتاجات: بعد العلاج بـ برازيكوانتيل، لم يكن لأوزان الكلاب، أجناسها، أعمارها، المدينة التي جاءت منها، الفترة الزمنية لطرد الديدان والحالة المعدية السابقة في أي من مراحل أخذ العينات أي تأثير على معدل انتشار عودة العدوى في المنطقة عموماً. كانت الاختلافات بين معدلات انتشار عودة العدوى على الأغلب بسبب زخم العدوى الموسمي لأنواع المشوّكات في المنطقة. يجب اعتبار بداية الشتاء، والربيع، وبداية الصيف مواسم مهمة لأنسب تدخل لطرد الديدان في هذه المجتمعات التبتية.

Translated from English version into Arabic by Muther J. Alohmayed, through

TRANSLATORS
WITHOUT BORDERS

四川省藏区家犬棘球绦虫再感染的季节性规律研究及其对包虫病防控的意义

王谦，喻文杰，钟波，尚婧晔，黄亮，亚力山大·马斯汀，仁青彭错，黄燕，张光葭，何伟，帕特里克·季洛杜，伍卫平，菲利普· S·克雷格

摘要

引言：四川省藏区是人群囊型包虫病和泡型包虫病的高度流行区。研究显示，该地区包虫病的主要传染源是家犬。观察发现，家犬在早冬和春天更易于获得棘球属绦虫的动物中间宿主，包括家畜内脏和小型哺乳

动物。因此，我们推测，犬的棘球绦虫感染风险在这两个季节会有显著升高，并以此为依据开展了犬的棘球绦虫再感染研究。

方法：本研究中的家犬粪便样本分别于 2009 年的 4 月（春天）、7 月（早夏）、9 月/10 月（秋天/早冬）、12 月（冬天）和 2010 年的 4 月（春天）采集于甘孜藏族自治州（中国四川）的 7 个乡（镇），通过粪抗原 ELISA 检测确定其棘球绦虫感染情况。每轮采样后（共 5 轮），给狗喂食吡喹酮以驱除犬体内感染的所有绦虫。详细记录每只家犬的性别、年龄和体重信息。研究中涉及的数据均值和率的比较，以及感染状态相关因素的确认采用 T 检验、Fisher 精确检验、泊松回归和 logistic 回归进行分析。

结果：研究中纳入的家犬中母犬比例明显低于公犬，母犬(22.78%)的粪 ELISA 基线感染率显著高于公犬(11.88%)。总的来说，犬的体重、性别、年龄、来源县和以前任何一次感染状态对犬的再感染都没有影响。没有证据显示本研究中不同的驱虫时间间隔对犬的再感染率有任何的影响。犬的棘球绦虫再感染率在 2009 年春天和早夏，以及 2009 年 9 月/10 月和 12 月间的早冬显著的高于其它时间点，提示以上季节犬棘球绦虫的感染压力高于其它的季节。

结论：总的来说，该地区犬的体重、性别、年龄、来源县、驱虫时间间隔和以前感染状态对再感染没有影响。犬再感染率的差异很可能来源于当地棘球属绦虫在不同季节表现出的不同的感染压力。早冬、春天和早夏应该是该藏区犬驱虫治疗效果最佳的重要季节。

Translated from English version into Chinese by Jing-Ye Shang

Caractère saisonnier des réinfections par *Echinococcus* chez les chiens domestiques des communautés tibétaines du Sichuan, en Chine, et ses répercussions pour le contrôle

Qian Wang, Wen-Jie Yu, Bo Zhong, Jing-Ye Shang, Liang Huang, Alexander Mastin, RENQINGPENGCUO, Yan Huang, Guang-Jia Zhang, Wei He, Patrick Giraudoux, Wei-Ping Wu, Philip S. CRAIG

Résumé

Contexte : L'échinococcose cystique (EC) et l'échinococcose alvéolaire (EA) chez l'homme sont des maladies fortement endémiques au sein des populations tibétaines de la province du Sichuan. Des études antérieures dans la région ont démontré que le chien domestique était la source majeure d'infection pour l'homme, et des observations ont montré que le chien domestique était plus susceptible d'entrer en contact avec les hôtes intermédiaires d'*Echinococcus* spp. : les viscères du bétail domestique (EC) et les petits mammifères (EA), au début de l'hiver et à nouveau au printemps. Nous avons émis l'hypothèse qu'il y aurait de ce fait une augmentation significative du risque d'infection chez les chiens avec l'*Echinococcus* spp. pendant ces deux saisons, et nous avons mené une étude sur la réinfection afin d'approfondir la recherche.

Méthodes : Des échantillons de fèces de chiens domestiques ont été prélevés dans sept subdivisions administratives de la préfecture autonome tibétaine de Garzê (province du Sichuan, Chine) et le statut infectieux d'*Echinococcus* spp. a été déterminé en utilisant le test copro-antigène ELISA. Les échantillons ont été prélevés sur les chiens en avril (printemps), en juillet (début de l'été), en septembre-octobre (automne-début de l'hiver) et en décembre (hiver) pour l'année 2009 ; et en avril (printemps) 2010. Les chiens ont été traités par praziquantel après chacun des cinq prélèvements d'échantillons pour éliminer tous ténias. Des informations sur le sexe, l'âge et le poids du chien ont également été recueillies. Le test t de Student, le test exact de Fisher, la régression de Poisson et la régression logistique ont été utilisés pour comparer les moyennes et les prévalences, et identifier les facteurs associés au statut infectieux.

Résultats : La proportion des femelles était significativement plus basse que celle des mâles ; les femelles

présentaient une prévalence significativement plus élevée (22,78 %) sur la base du test copro-ELISA que les mâles (11,88 %). Le poids, le sexe, l'âge, le comté d'origine et les antécédents de statut infectieux à tout point de prélèvement n'avaient aucune influence sur la prévalence de réinfection en général. La régression de Poisson n'a démontré aucune influence significative sur la prévalence de réinfection en raison de périodes de traitement vermifuge/échantillonnage différentes. Les chiens ont présenté des prévalences de réinfection significativement plus élevées au printemps et au début de l'été 2009, ainsi qu'au début de l'hiver, entre septembre octobre et décembre 2009, indiquant une pression d'infection plus élevée au cours de ces saisons que le reste de l'année.

Conclusion : En se basant sur le traitement par praziquantel, le poids, le sexe, l'âge et le comté d'origine du chien, la période de traitement vermifuge et les antécédents de statut infectieux à tout point de prélèvement n'avaient aucune influence sur la prévalence de réinfection dans la région en général. Les différences entre prévalences de réinfection étaient probablement dues à la saisonnalité de la pression d'infection d'*Echinococcus* spp. dans la région. Le début de l'hiver, le printemps et le début de l'été devraient être des saisons importantes pour un traitement vermifuge optimal chez les chiens dans ces communautés tibétaines.

Translated from English version into French by Adeline Brahim, through

TRANSLATORS WITHOUT BORDERS

Характер сезонных изменений повторного заражения *Эхиноккоком* собак, принадлежащих тибетским общинам провинции Сычуань, Китай, и его использование при контроле

Киан Ванг, Вен-Йие Йу, Бо Жонг, Жинг-Йе Шанг, Лианг Хуанг, Александр Мастин, Ренкингпенгкуо, Йан Хуанг, Гуанг-Йиа Жанг, Вей Хе, Патрик Жирадо, Вей-Пинг Ву, Филипп С. Крэйг

Краткий обзор

Исходные данные: Человеческий эхинококкоз мочевого пузыря (CE) и альвеолярный эхинококкоз (AE) - эндемическое заболевание тибетских общин провинции Сычуань. Предыдущее исследование, проведенное в регионе, показало, что домашние собаки были главным источником инфицирования человека, а наблюдения показали, что домашние собаки имеют больше доступа к непосредственным организмам-носителям *Эхинококка*: к домашнему скоту (CE внутренних органов) и к мелким млекопитающим (AE) ранней зимой и повторно весной. Мы предположили, что вследствие этого значительно возрастает риск собачьего заражения *Эхинококком* в оба эти периода, и провели изучение повторного заражения для подтверждения этой гипотезы.

Методы: Были собраны образцы фекалий от домашних собак в семи населенных пунктах Гардзе-Тибетского автономного округа (провинция Сычуань, Китай), и статус заражения видами *Эхинококка* был определен с помощью использования иммуно-ферментного анализа калового антигена. Собаки были отобраны в апреле (весна), июле (ранее лето), сентябре/октябре (осень/ранняя зима), декабре (зима) 2009 года и в апреле 2010. Собакам был введен Празиквантел после сбора каждого из пяти образцов для того, чтобы уничтожить всех ленточных червей. Также была собрана информация о поле, возрасте и массе тела собак. Для сравнения средств и уровня распространения, а также для определения факторов, соотносимых со статусом заражения, были использованы критерий Стьюдента, точный критерий Фишера, регрессия Пуассона и логистическая

регрессия.

Результаты: Количественное соотношение собак женского пола было значительно ниже, чем мужского; у собак женского пола значительно более высокое (22.78%) распространение первичного иммуноферментного твердофазного анализа кала, чем у собак мужского пола (11.88%). В целом, на распространенность повторного заражения не влияли вес тела, пол, возраст, район или предыдущий инфекционный статус собак ни в одном месте отбора проб. Регрессия Пуассона не показала значительного влияния на распространенность повторного заражения разных промежутков времени между дегельминтацией и отбором проб. Собаки показали значительно более высокую распространенность повторного заражения весной и ранним летом 2009 года, а также ранней зимой между сентябрем/октябрем и ноябрем 2009 года, что предполагает более высокое инфекционное давление в эти периоды года по сравнению с другими.

Заключение: в целом на распространенность повторного заражения в указанном районе не влияли последующее лечение Празиквантелом, а также вес тела, пол, возраст, район или предыдущий инфекционный статус собак ни в одном из мест отбора проб. Предположительно, разница между распространенностью повторного заражения вызвана сезонностью инфекционного давления видом *Эхинококка* в указанном районе. Ранняя зима, весна и ранее лето являются важными периодами года для оптимальной дегельминтации собак в указанных Тибетских общинах.

Translated from English version into Russian by Natallia Lupik, through

TRANSLATORS WITHOUT BORDERS

Patrón estacional de la reinfección por *Echinococcus* en perros domésticos en comunidades tibetanas de Sichuan (China) y sus implicaciones para el control

Qian Wang, Wen-Jie Yu, Bo Zhong, Jing-Ye Shang, Liang Huang, Alexander Mastin, RENQINGPENGCUO, Yan Huang, Guang-Jia Zhang, Wei He, Patrick Giraudoux, Wei-Ping Wu, Philip S. CRAIG

Resumen

Información de referencia: La equinococosis cística humana (EC) y la equinococosis alveolar (EA) son muy endémicas en las comunidades tibetanas de la provincia china de Sichuan. La investigación realizada anteriormente en la región indicaron que el perro doméstico era la principal fuente de infección para las personas y las observaciones indicaron que el perro doméstico podría tener más acceso a huéspedes intermedios de *Echinococcus* spp: tanto vísceras de ganado doméstico (EC) como pequeños mamíferos (EA), a principios del invierno y de nuevo en primavera. Nuestra hipótesis era que habría por tanto un significativo aumento del riesgo de infección canina con *Echinococcus* spp. en estas dos estaciones y llevamos a cabo un estudio de la reinfección para investigarlo con mayor detalle.

Métodos: Se recogieron muestras fecales de perros domésticos en siete municipios de la Prefectura Autónoma de Ganze Tíbet (provincia de Sichuan, China) y se determinó el estado de la infección de *Echinococcus* spp. utilizando el coproantígeno ELISA. Las muestras de los perros se tomaron en abril (primavera), julio (principios de verano), septiembre/octubre (otoño/principio de invierno) y diciembre (invierno) de 2009; y en abril (primavera) de 2010. Se trató a los perros con praziquantel tras cada una de las cinco recogidas de muestras para eliminar las tenias que

pudiera haber. También se recogió información sobre el sexo, edad y peso del perro. Se aplicaron la prueba t de Student, la prueba exacta de Fisher, la regresión de Poisson y la regresión logística para comparar media y prevalencias, así como para identificar los factores asociados al estado de la infección.

Resultados: La proporción de hembras de perro fue significativamente más baja que la de machos; las hembras presentaron una prevalencia significativamente más alta (22,78%) de copro-ELISA que los machos (11,88%). El peso, sexo, edad, comarca e infecciones anteriores en algún momento de la fase de muestreo no influyeron sobre la prevalencia de la reinfección en general. La regresión de Possion no halló una influencia significativa sobre la prevalencia de la reinfección debida a diferentes plazos de tiempo de eliminación de parásitos/muestreo. Los perros mostraron unas prevalencias de reinfección significativamente más elevadas en primavera y a principios del verano de 2009 y a principios del invierno entre septiembre/octubre y diciembre de 2009, lo cual sugiere una mayor presión de la infección en estas estaciones si comparan con otras.

Conclusión: Tras el tratamiento con praziquantel, el peso, sexo, edad del perro, comarca, tiempo transcurrido desde la eliminación de parásitos y el estado de infecciones anteriores en ningún momento del muestreo influyeron sobre la prevalencia de la reinfección en la región en general. Las diferencias entre las prevalencias de reinfección se debieron probablemente a la estacionalidad de la presión de infección de *Echinoccocus* spp. en la región. Principios del invierno, primavera y principios de verano deberían ser estaciones importantes para una óptima eliminación de los parásitos en estas comunidades tibetanas.

Translated from English version into Spanish by SergioLorenzi, through

TRANSLATORS
WITHOUT BORDERS
